# Talking Head Generation Through Generative Models and Cross-Modal Synthesis Techniques

**DOI:** 10.3390/jimaging12030119

**Published:** 2026-03-10

**Authors:** Hira Nisar, Salman Masood, Zaki Malik, Adnan Abid

**Affiliations:** 1 Department of Data Science, Faculty of Computing and Information Technology, University of the Punjab, Lahore 54590, Pakistan; msdsf23m004@pucit.edu.pk; 2 Dyads Consulting, 16734 NW Lynch LN, Portland, OR 97229, USA; salmanmsd@gmail.com; 3 Department of Marketing and Business Analytics, East Texas A & M University, Commerce, TX 75429, USA; zaki.malik@tamuc.edu

**Keywords:** talking head generation, audio–visual synthesis, facial animation, deep learning, generative AI, GANs

## Abstract

Talking Head Generation (THG) is a rapidly advancing field at the intersection of computer vision, deep learning, and speech synthesis, enabling the creation of animated human-like heads that can produce speech and express emotions with high visual realism. The core objective of THG systems is to synthesize coherent and natural audio–visual outputs by modeling the intricate relationship between speech signals, facial dynamics, and emotional cues. These systems find widespread applications in virtual assistants, interactive avatars, video dubbing for multilingual content, educational technologies, and immersive virtual and augmented reality environments. Moreover, the development of THG has significant implications for accessibility technologies, cultural preservation, and remote healthcare interfaces. This survey paper presents a comprehensive and systematic overview of the technological landscape of Talking Head Generation. We begin by outlining the foundational methodologies that underpin the synthesis process, including generative adversarial networks (GANs), motion-aware recurrent architectures, and attention-based models. A taxonomy is introduced to organize the diverse approaches based on the nature of input modalities and generation goals. We further examine the contributions of various domains such as computer vision, speech processing, and human–robot interaction, each of which plays a critical role in advancing the capabilities of THG systems. The paper also provides a detailed review of datasets used for training and evaluating THG models, highlighting their coverage, structure, and relevance. In parallel, we analyze widely adopted evaluation metrics, categorized by their focus on image quality, motion accuracy, synchronization, and semantic fidelity. Operating parameters such as latency, frame rate, resolution, and real-time capability are also discussed to assess deployment feasibility. Special emphasis is placed on the integration of generative artificial intelligence (GenAI), which has significantly enhanced the adaptability and realism of talking head systems through more powerful and generalizable learning frameworks.

## 1. Introduction

Talking Head Generation, a pivotal technology in computer graphics and artificial intelligence, has witnessed remarkable advancements in recent years. This technology facilitates the creation of animated representations of human heads capable of speech and emotional expression. It exhibits a wide range of applications, encompassing both advantageous uses such as virtual assistants/avatars [1] and video dubbing [2] as well as potentially detrimental applications such as deepfake technology. The ability to generate realistic talking heads is not only pivotal for enhancing human–computer interaction and providing immersive experiences in virtual and augmented reality environments but also holds significant promise in entertainment and accessibility technologies [3].

In this review paper, we aim to provide a comprehensive overview of Talking Head Generation, exploring its methodologies, applications, datasets, evaluation metrics, and operating parameters. Unlike prior surveys, our work introduces a novel taxonomy and unifying framework that integrates methodological, data-driven, and deployment perspectives, offering deeper cross-domain insights and a critical comparative analysis of existing approaches. By synthesizing existing research, we seek to identify current trends and potential future directions in this dynamic and rapidly evolving field.

The scope of Talking Head Generation encompasses various domains and fields, each leveraging this technology to address specific challenges and create new opportunities. In the realm of virtual assistants and avatars, realistic talking heads enhance user engagement and provide a more natural interaction experience. This is particularly evident in customer service, where virtual agents can offer personalized support and improve customer satisfaction. In the entertainment industry, talking head generation is revolutionizing the creation of animated films, video games, and virtual reality experiences by enabling lifelike character animations and realistic dubbing for multilingual content. Furthermore, in the education sector, talking head technology enables the creation of interactive and engaging educational content, facilitating better comprehension and retention among students. From a financial viewpoint, companies are investing in this technology to develop advanced marketing strategies, create engaging advertising campaigns, and produce high-quality content that resonates with audiences. For instance, personalized talking head avatars can enhance customer experiences in e-commerce by providing tailored recommendations and virtual try-ons, driving higher conversion rates.

The impact of Talking Head Generation on society is profound. It has opened new avenues for accessibility technologies, aiding individuals with hearing impairments through realistic sign language interpreters and lip-reading aids. It holds potential in healthcare too, particularly in telemedicine, where virtual doctors and therapists can provide remote consultations with a human touch. This technology also enables the preservation and digital resurrection of historical figures, allowing for interactive educational experiences and cultural preservation. However, the ethical implications of this technology cannot be overlooked, especially concerning deepfake applications, which can be misused to spread misinformation, manipulate public opinion, and violate privacy. Addressing these ethical challenges is crucial for the responsible development and deployment of talking head technology. In the following sections, we will expand on exactly what this technology entails and why it matters.

### 1.1. What Is Talking Head Generation

Talking Head Generation refers to the technology and methodologies used to create animated representations of human heads that can speak and express emotions. Talking-head video generation aims to produce realistic animated videos of a speaking person, mirroring the characteristics of an input source [4].

**Figure 1:**
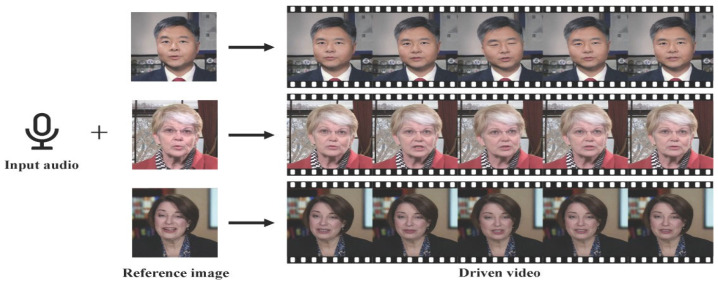
Talking Head Generation.

**Figure 2:**
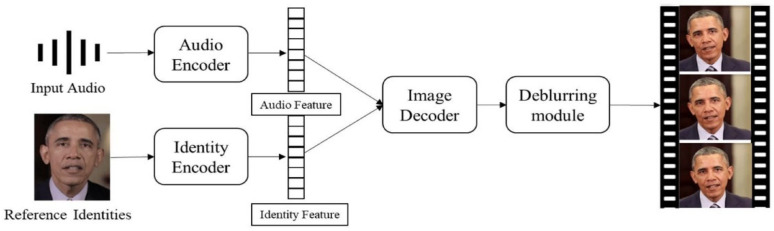
Overview of Speech to Video Process.

This basic process of audio and image synthesis is shown in Figure 1 [5]. This process involves the synthesis of audio and visual data to produce realistic, dynamic facial animations that can be used in a variety of applications [6]. Key components of this process include capturing the nuances of human facial expressions, lip movements synchronized with speech, and conveying emotions authentically. Recent advancements in deep learning, computer vision, and natural language processing have significantly enhanced the capabilities of talking head generation systems, making them more realistic and versatile. This is shown in Figure 2 [3].

### 1.2. Why Is Talking Head Generation Important

The ability to generate realistic talking heads is crucial for creating virtual humans, enhancing human–computer interaction, and providing more immersive experiences in virtual and augmented reality environments. This technology enables the development of sophisticated virtual assistants that can engage users in natural and intuitive conversations, thereby improving user experience and satisfaction. In the entertainment industry, talking head generation is essential for creating believable and expressive characters in animated films, video games, and virtual reality applications, enhancing storytelling and audience engagement. Additionally, talking head technology plays a vital role in accessibility, providing tools such as sign language interpreters and lip-reading aids [7] that improve communication for individuals with hearing impairments. Furthermore, it holds promise in education, allowing for the creation of interactive and personalized learning experiences that can adapt to individual student needs and preferences.

### 1.3. Our Contributions

In this paper, we outline our primary contributions as follows:

- We offer a comprehensive literature review spanning the diverse domains which are used to perform talking-head generation.
- We introduce novel taxonomies and a unifying framework aimed at categorizing the methodologies utilized for performing talking-head generation, providing deeper cross-domain integration than prior surveys.
- We compile an overview of the prevalent datasets and evaluation metrics typically employed in the assessment of talking-head generation models, while analyzing their applicability, limitations, and interrelations.
- We explore future research directions, highlighting challenges and potential advancements in talking-head generation, with an emphasis on practical deployment considerations and trade-offs between realism, temporal consistency, and computational cost.

## 2. Related Work

The field of Talking Head Generation has garnered significant attention, leading to a variety of review articles that summarize the progress and advancements made thus far. These reviews provide valuable insights and lay the groundwork for further research and development. Below, we summarize some of the notable contributions in this domain to clarify the scope and focus of prior surveys in relation to the present work.

A review article [3] focuses on the comprehensive framework for human–computer interaction involving virtual humans, particularly emphasizing speech recognition, text-to-speech, dialogue systems, and virtual human generation. It categorizes talking-head video generation models within the virtual human deep generation framework and provides a systematic review of technological advancements and trends over the past five years, highlighting critical works and datasets. In contrast, our survey places primary emphasis on talking head generation itself, with deeper technical analysis of generation models and system-level design choices.

Another survey [8] categorizes talking head generation methods into image-driven, audio-driven, video-driven, and other approaches such as Neural Radiance Fields (NeRF) and 3D-based methods [9]. It provides an in-depth analysis of each method’s contributions, strengths, and limitations, and compares publicly available models based on inference time and human-rated quality. While this work focuses on method categorization and runtime comparison, our manuscript extends the analysis by systematically comparing approaches in terms of realism, temporal consistency, computational cost, data requirements, and generalization ability.

An important study [6] focuses on synthesizing natural talking human faces using deep learning methods such as Convolutional Neural Networks (CNNs), GANs, and NeRF. It reviews these methods, discusses implementation challenges, and highlights open research issues across application domains. Our survey complements this by incorporating recent GenAI-driven architectures and by explicitly analyzing deployment-related factors such as latency, frame rate, and real-time feasibility.

Moreover, a study [4] addresses limitations in performance evaluation by proposing standardized benchmarks and new evaluation metrics for talking-head video generation. It conducts a thorough analysis of state-of-the-art approaches. Building on this, our review integrates evaluation metrics with model taxonomy and dataset characteristics, offering a unified perspective across modeling, data, and assessment dimensions.

While these review articles provide valuable insights into specific aspects of talking-head generation, the present manuscript distinguishes itself by offering an integrated and holistic perspective that unifies methodological taxonomy, critical comparative analysis, dataset review, evaluation metrics, and deployment insights. This approach provides a unique perspective on cross-domain integration and model trade-offs, supporting both researchers and practitioners in understanding not only how existing methods work, but also their practical implications for real-world applications.

## 3. Discussion

**Figure 3:**
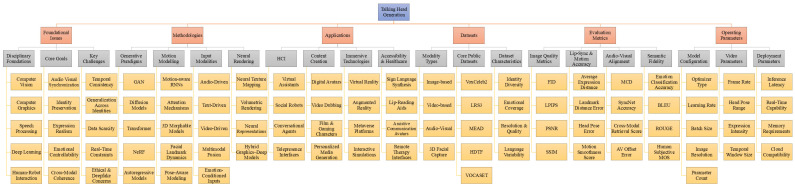
Unified hierarchical taxonomy of Talking Head Generation.

Talking Head Generation is a rapidly evolving field that integrates advances across multiple disciplines to create realistic and expressive virtual human heads capable of speech and emotional expression. The underlying technologies draw upon developments in computer vision, deep learning, virtual reality, and robotics, each contributing essential capabilities to visual realism, motion modeling, interaction, and embodiment. As progress continues across these domains, the scope of talking head applications has expanded significantly, enabling enhanced human–computer interaction, immersive virtual environments, improved communication in robotics, and increasingly sophisticated virtual assistants. To provide a coherent and structured understanding of this diverse research landscape, this section now introduces a unified hierarchical taxonomy that consolidates the previously discussed categorizations into a single framework. The unified taxonomy integrates disciplinary foundations, methodological approaches, application scenarios, datasets, evaluation metrics, and system parameters within a common hierarchical structure (Figure 3). This consolidated view captures the full Talking Head Generation pipeline while preserving the distinct analytical roles of each component, thereby offering a comprehensive and interpretable overview of the field.

### 3.1. Overview of Domains

**Figure 4:**
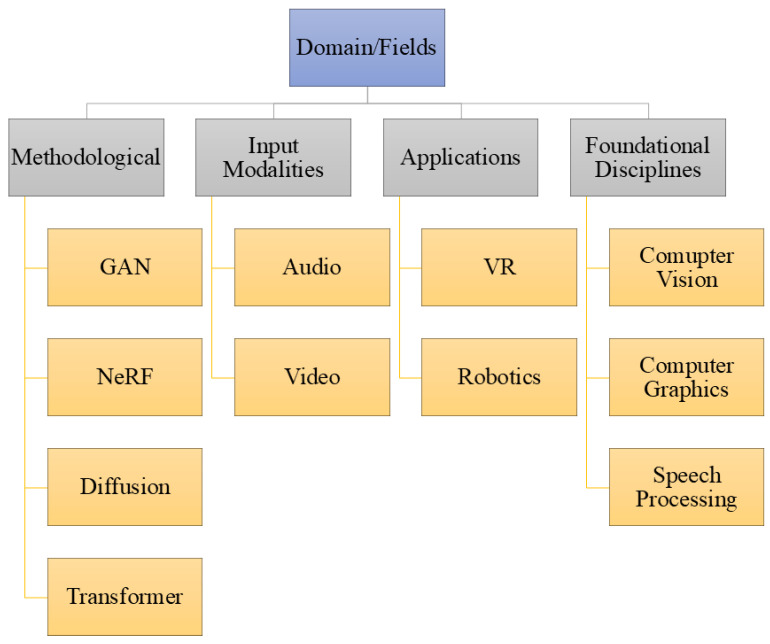
Breakdown of various domains.

Early research in Talking Head Generation (THG) can be traced to disciplinary foundations such as computer vision, computer graphics, and deep learning. However, contemporary THG systems are characterized by tightly integrated neural representation learning, geometric modeling, and rendering pipelines. Accordingly, we reorganize this section around the core modeling paradigms that underpin modern THG systems, while application domains such as Virtual Reality and Robotics are discussed separately as deployment contexts (Figure 4).

#### 3.1.1. GAN-Based Talking Head Generation

Generative Adversarial Networks (GANs) represent one of the earliest and most influential paradigms in neural talking head synthesis. GAN-based methods [10,11,12,13,14] explicitly optimize data distribution learning to generate high-frequency facial details and photorealistic outputs. While these approaches achieve strong visual realism, they often suffer from training instability and temporal inconsistency in long sequences.

HeadGAN [15] enhances realism by integrating 3D facial representations, though at the cost of increased model complexity. Similarly, attention-based GAN frameworks [13,16] enable strong cross-modal alignment and speaker-specific control, albeit requiring large-scale datasets and significant computational resources.

Audio-driven single-image synthesis approaches [17,18] demonstrate strong visual quality but struggle with long-term temporal coherence. Personalized and multi-person GAN systems [17,19,20,21] improve identity preservation and expressiveness, though scalability remains a challenge.

#### 3.1.2. NeRF-Based and Neural Rendering Approaches

Neural Radiance Field (NeRF)-based talking head systems [22,23,24,25] represent a significant methodological shift toward implicit neural representations and differentiable rendering. These approaches combine neural networks with volumetric rendering techniques, enabling superior view consistency, fine-grained facial detail, and controllable editing capabilities.

Neural rendering-based one-shot methods [26,27,28] emphasize controllability and reconstruction fidelity rather than explicit distribution modeling. Weakly supervised 3D reconstruction methods [28] enhance geometric consistency but incur higher computational cost.

Data-driven photorealistic head modeling from phonetic transcripts [29] and 3D morphable model-based methods [30] further strengthen geometric realism but require careful preprocessing and high-quality training data. SLIGO [31] captures complex facial dynamics and emotions through stochastic latent modeling but remains computationally demanding.

Although NeRF-based and neural rendering approaches deliver high perceptual quality and structural coherence, their reliance on complex rendering pipelines limits real-time scalability.

#### 3.1.3. Diffusion-Based and Transformer-Based Methods

Diffusion-based talking head generation has recently emerged as a powerful alternative to GAN-based synthesis. VLOGGER [29] leverages diffusion models to achieve strong identity preservation and temporal consistency, though with increased computational overhead. Diffusion-based motion synthesis techniques [32,33,34,35,36,37] emphasize disentanglement of identity, speech content, and style while improving perceptual quality and synchronization.

Transformer-based cross-domain reenactment methods [38] generalize effectively across domains but face challenges under extreme poses. Motion-aware recurrent neural networks (RNNs) [10,39] model speech-aligned head dynamics effectively but provide limited appearance control. Wav2Lip prioritizes lip-sync accuracy, sometimes at the expense of holistic facial motion modeling.

**Figure 5:**
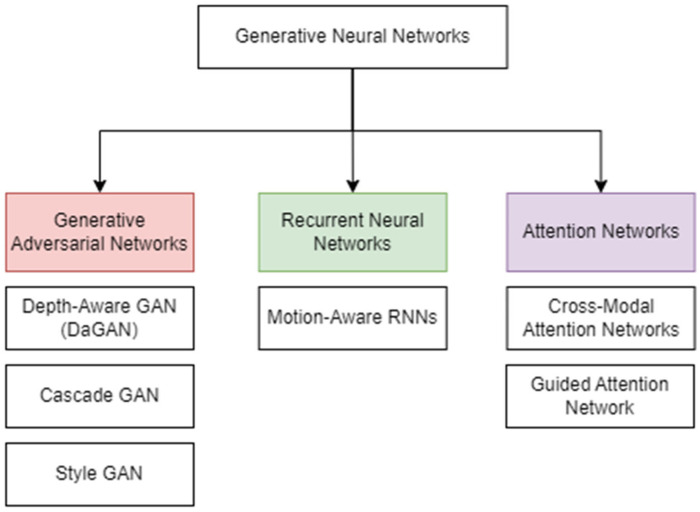
Generative Deep Learning Techniques.

Keypoint-based and bandwidth-efficient models reduce transmission requirements but may sacrifice fine-grained texture detail. Attention-driven frameworks [13,16] strengthen cross-modal alignment and speaker-specific control while remaining computationally intensive. Collectively, diffusion and transformer-based approaches reflect the ongoing shift toward large-scale generative modeling and multimodal integration, balancing visual fidelity, temporal coherence, and controllability (Figure 5). Also, the summary of Deep Learning Approaches in THG is shown in Table 1.

### 3.2. Input Modalities in THG

Modern THG systems are commonly categorized based on their conditioning modality.

#### 3.2.1. Audio-Driven Generation

Audio-driven methods [10,17,18,39] model speech-to-motion alignment, focusing on lip synchronization, head dynamics, and prosody-consistent facial expression. While achieving strong synchronization, these systems may struggle with long-term semantic coherence.

#### 3.2.2. Multimodal and Cross-Modal Fusion

Multimodal fusion approaches [38,40] integrate audio, visual, and auxiliary signals (e.g., eye tracking) to enhance realism and conversational naturalness. Codec Avatar-based systems [40] achieve low-latency and immersive facial animation but depend on specialized hardware.

### 3.3. Application Contexts: Virtual Reality and Robotics

#### 3.3.1. Virtual Reality Deployment

VR-oriented talking head systems prioritize immersion and real-time responsiveness. Real-time avatar generation methods [40,41,42,43,44] balance latency and realism to support interactive environments. Emotion-focused datasets and generation frameworks [1] enhance stylized avatar interactivity.

Although NeRF-based and diffusion-driven models [23,24,25] provide superior visual fidelity and editability, their computational demands limit widespread real-time deployment.

#### 3.3.2. Robotics and Human–Robot Interaction

Robotics represents a specialized application domain rather than a methodological driver. Speech-driven animatronic facial animation [45] demonstrates precise real-time mechanical control of robotic facial expressions. However, such systems primarily rely on advances in neural modeling and computer vision rather than introducing independent modeling paradigms. Hardware constraints and mechanical design considerations further limit scalability.

### 3.4. Overview of Application Approaches Used in Talking Head Generation

**Figure 6:**
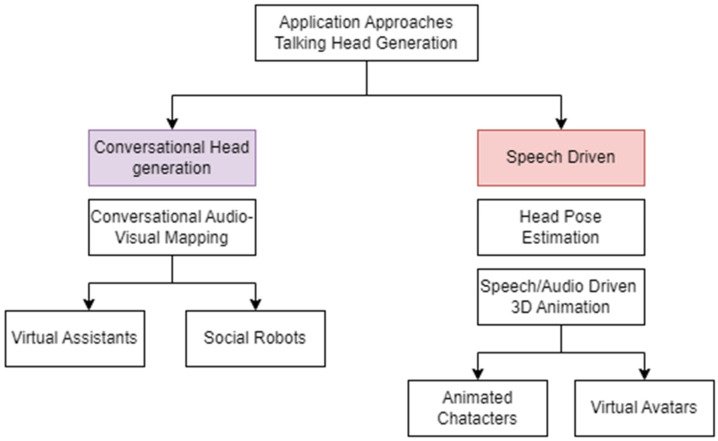
Application Approaches in Talking Head Generation.

As shown in Figure 6, application approaches broadly fall into conversational head generation and speech-driven animation. Conversational heads incorporate NLP and dialogue management to enable context-aware, multi-turn interaction, achieving richer engagement at the cost of higher latency and system complexity. Speech-driven methods focus on accurate audio–visual synchronization, typically offering higher visual fidelity and lower latency but reduced contextual awareness.

#### 3.4.1. Conversational Head Generation

Recent advances in conversational head generation demonstrate trade-offs between realism, interactivity, and scalability. The winning approach in the ACM Multimedia ViCo 2023 Conversational Head Generation Challenge [42] combines audio-driven 3DMM parameter prediction with neural rendering to produce realistic lip-sync videos, achieving strong visual quality but relying on carefully curated data and rendering pipelines. Gesture prediction from speech in unconstrained settings [46] improves conversational naturalness by mapping raw audio to pose sequences using unlabeled data; however, such methods may exhibit temporal inconsistency or reduced robustness under noisy audio conditions. While large-scale person-specific gesture datasets further enhance realism and engagement, these approaches often increase training complexity and may struggle with generalization across speakers or interaction styles.

#### 3.4.2. Speech-Driven Animation

Speech-driven animation methods exhibit diverse design choices with clear trade-offs among realism, controllability, and data efficiency. Transformer-based models such as FaceFormer [47,48] achieve accurate lip synchronization and smooth motion but require large-scale training data and may introduce latency. Emotion-aware systems like EVP [49] and Style2Talker improve expressiveness by disentangling emotion and speech, though they can suffer from emotional inconsistency when emotion labels are ambiguous. Audio-driven personalized animation systems [50,51] and SadTalker [52] enhance realism and head pose control via 3D motion modeling but may experience identity drift or increased computational cost. Diffusion-based approaches, including DiffPoseTalk [53] and DREAM-Talk [54], generate diverse and expressive animations with strong temporal coherence, albeit at the expense of higher inference time. Lightweight or one-shot methods such as VectorTalker [55] improve controllability and efficiency but may sacrifice fine-grained realism. Other frameworks, including Flow2Flow, VividTalker [56], and methods incorporating lipread loss and adaptive modulation [57], balance identity preservation, motion accuracy, and perceptual quality, yet often depend on complex architectures and extensive supervision. Collectively, these methods highlight key trade-offs in speech-driven animation, where gains in realism and expressiveness frequently come at the cost of latency, controllability, or data requirements, influencing their suitability for different real-world applications.

### 3.5. Overview of Datasets

**Figure 7:**
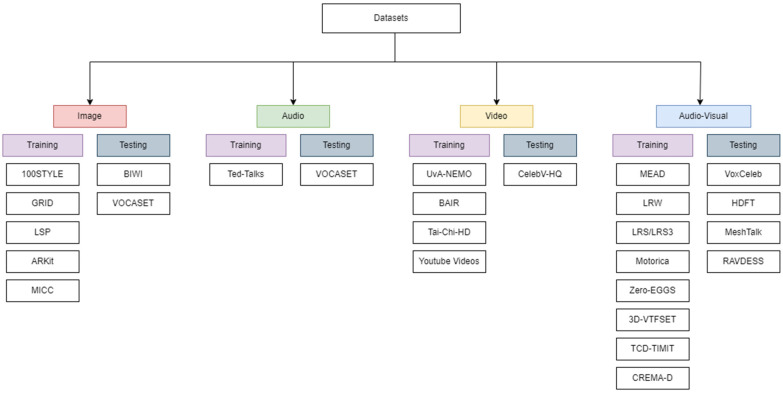
Some of the datasets used in Talking Head Generation.

Datasets play a central role in advancing talking head generation by providing audio–visual, geometric, and emotional data required for training and evaluation. Existing datasets vary widely in scale, modality, realism, and annotation richness, which directly affects model generalization, expressiveness, and evaluation reliability. Rather than serving all tasks uniformly, different datasets are better suited to specific objectives such as lip synchronization, emotion modeling, 3D facial animation, or robustness to in-the-wild conditions. Figure 7 provides a high-level overview of commonly used datasets and their roles.

#### 3.5.1. Division into Training and Evaluation Sets

Training Datasets: Training datasets for Talking Head Generation (THG) emphasize diversity, realism, multimodality, and expressive richness to support robust model generalization. Motion- and pose-focused datasets such as 100STYLE [36] and LSP [42] enable stylistic variation and improved motion learning. Audio–visual speech datasets including GRID [19,58,59], LRW [60], LRS/LRS3 [61], and TED-Talks [62] are widely adopted for lip synchronization and speech-driven animation. Emotion-aware datasets such as MEAD [61,62], CREMA-D [19], and Emotional ARKit [54] facilitate affective modeling and expressive facial synthesis.

For high-fidelity geometric consistency and rendering supervision, datasets including MICC Florence & FaceWarehouse [63], UvA-NEMO [64], 3D-VTFSET [56], and TCD-TIMIT [61] provide detailed facial structure and multimodal annotations. Although not specifically designed for talking head synthesis, motion-centric datasets such as BAIR Robot Pushing [64], Tai-Chi-HD [64], Zero-EGGS [36], and Motorica Dance [36] contribute valuable insights into temporal dynamics and motion coherence.

Testing/Benchmark Datasets: Evaluation datasets prioritize controlled settings, accurate annotations, and reproducibility. VOCASET [9] and BIWI [9,47] are commonly used for benchmarking lip motion and head pose estimation, respectively. MeshTalk [9] supports detailed 3D mesh-based evaluation, while HDTF [5,20,22,37,65] enables high-resolution visual benchmarking. Large-scale datasets such as CelebV-HQ [13] and VoxCeleb1/2 [58,62,64,66] serve as de facto benchmarks for generalization and scalability, while RAVDESS [66] is widely used for evaluating emotional accuracy.

#### 3.5.2. Important Datasets and Their Stats

Key dataset statistics—including modality, duration, number of subjects, presence of head motion, and collection environment—are summarized in Table 2, enabling direct comparison and informed dataset selection.

#### 3.5.3. Dataset Thoroughness (Covering Edge Cases)—VoxCeleb2

Large-scale, in-the-wild datasets such as VoxCeleb2 [58,62,64,66] and CelebV-HQ [43] offer extensive coverage of speakers, poses, lighting conditions, and recording environments. Their diversity and authenticity make them particularly valuable for stress-testing models against real-world variability and edge cases, improving robustness and generalization.

#### 3.5.4. Segregation into Image/Video/Audio Sets

Table 3 summarizes representative tools and algorithms that have been evaluated using widely adopted benchmark datasets such as VoxCeleb1/2 and CelebV-HQ.

#### 3.5.5. Segregation by Modality

Datasets used in talking head generation can be broadly grouped into image, video, audio, audio–visual, and emotional multimodal categories. This modality-based organization clarifies dataset suitability for tasks such as static identity modeling, temporal animation, speech-driven synthesis, or emotion-aware generation, enabling more actionable dataset selection for specific applications.

### 3.6. Overview of Evaluation Metrics

**Figure 8:**
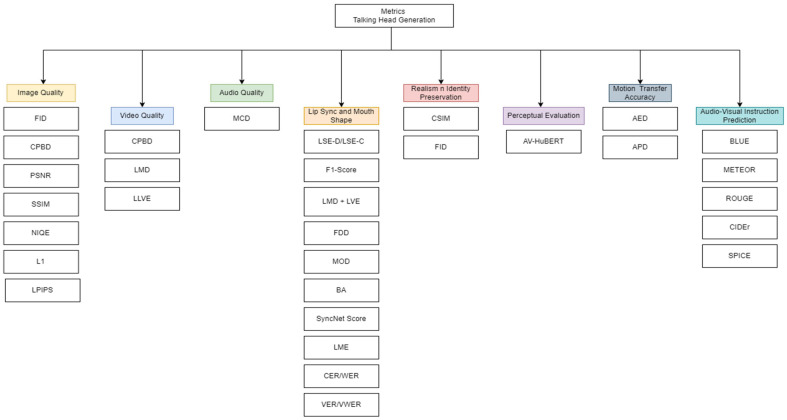
Breakdown of Evaluation Metrics. This figure illustrates the various evaluation metrics used in the study.

A high-level categorization of evaluation metrics used in Talking Head Generation (THG) is illustrated in Figure 8. Unlike conventional image or video synthesis tasks, THG requires simultaneous assessment of visual realism, temporal coherence, audio–visual synchronization, identity preservation, and perceptual naturalness, making metric selection non-trivial and task-dependent [67].

#### 3.6.1. Image Quality Metrics

Image-level metrics are widely used to assess frame-wise visual fidelity, but their suitability for THG varies. Fréchet Inception Distance (FID) [9] and LPIPS [38] are commonly adopted as perceptual metrics that correlate better with human judgments than pixel-wise measures. However, FID operates on global feature distributions and is insensitive to temporal artifacts and lip-sync errors, limiting its standalone interpretability for talking head videos. PSNR [38], SSIM [20], and L1 loss [13] remain useful for controlled reconstruction tasks but often correlate poorly with perceived realism in expressive facial animation. Metrics such as CPBD [13] and NIQE [42] provide complementary insights into sharpness and naturalness, yet they are sensitive to background texture and lighting variations common in in-the-wild THG datasets. Consequently, image metrics are most informative when used comparatively and in conjunction with motion- and synchronization-aware measures.

#### 3.6.2. Video Quality and Temporal Consistency

Temporal artifacts are particularly detrimental in THG, as even minor inconsistencies can disrupt perceived realism. Lip Landmark Distance (LMD) [66] and Lip Landmark Velocity Error (LLVE) [35] explicitly measure spatial accuracy and temporal smoothness of lip motion, making them more task-relevant than generic video metrics. While CPBD remains useful for detecting blur, it does not capture temporal jitter or motion drift. These limitations highlight the need to pair frame-based sharpness metrics with landmark-based temporal measures to properly evaluate talking head videos.

#### 3.6.3. Audio Quality and Audio–Visual Alignment

Audio quality is commonly evaluated using Mel Cepstral Distance (MCD) [28], which effectively measures spectral similarity between generated and reference speech. While MCD reflects intelligibility and acoustic fidelity, it does not account for perceptual synchronization with facial motion. To address this gap, multimodal perceptual metrics such as AV-HuBERT [28] have been introduced, providing a more holistic evaluation by jointly modeling audio and visual cues. Such metrics are particularly valuable for THG, where perceptual alignment often outweighs isolated audio or visual quality.

#### 3.6.4. Realism and Identity Preservation

Maintaining identity consistency is critical, especially in personalized and one-shot THG. The Cosine Similarity Identity Metric (CSIM) [13], often computed using ArcFace embeddings, directly measures identity preservation, while FID complements this by assessing distribution-level realism. However, identity metrics may fail to detect gradual identity drift over long sequences, underscoring the importance of combining identity scores with temporal and perceptual evaluations.

#### 3.6.5. Lip Synchronization and Mouth Shape Accuracy

Lip-sync accuracy is central to THG evaluation and is not adequately captured by generic image or video metrics. Specialized measures such as LSE-D, LSE-C, F1-score, Balanced Accuracy (BA), and SyncNet scores directly assess audio–visual alignment. Landmark-based metrics (LMD, LVE) further quantify spatial and temporal mouth dynamics, while perceptual measures such as Human Perceptual Distance (HPD) [5] better reflect subjective realism. Text-level metrics including CER, WER, VER, and VWER provide indirect evidence of synchronization quality by evaluating speech intelligibility and visual articulation consistency.

#### 3.6.6. Motion Transfer and Instruction-Level Evaluation

For expression and pose transfer, Average Expression Distance (AED) and Average Pose Distance (APD) are widely used to quantify motion accuracy but are sensitive to annotation noise and reference quality. In instruction- or text-conditioned THG, NLP metrics such as BLEU, METEOR, ROUGE, CIDEr, and SPICE are employed to assess semantic correctness of generated instructions. While effective for content alignment, these metrics do not directly measure visual realism and must be interpreted cautiously in multimodal settings.

#### 3.6.7. Qualitative and Human-Centered Evaluation

Despite extensive quantitative evaluation, qualitative metrics remain indispensable in THG. Subjective assessments of naturalness [30], clarity, expressiveness [41], and coherence [59] capture perceptual factors that are difficult to formalize but critical for user acceptance. Human studies are therefore essential for validating claims of realism and engagement.

#### 3.6.8. Metric Selection Rationale

No single metric sufficiently captures THG quality. Image metrics are effective for visual fidelity, landmark-based metrics for synchronization, motion metrics for temporal consistency, and perceptual evaluations for holistic realism. Table 4 summarizes the strengths, limitations, and recommended usage contexts of commonly used metrics, providing guidance for selecting appropriate evaluation protocols based on specific THG task characteristics.

### 3.7. Overview of Operating Parameters

**Figure 9:**
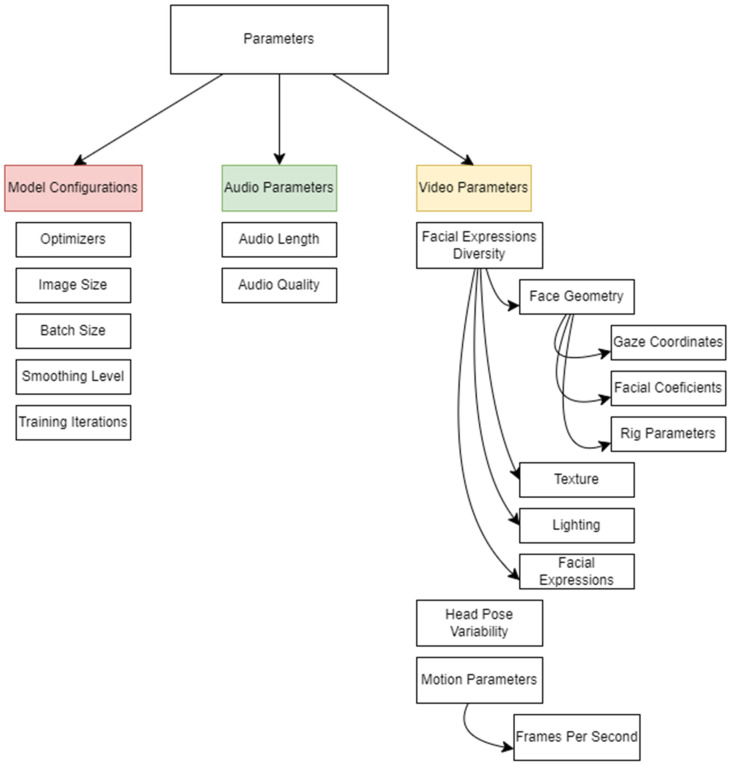
Breakdown of Operating Parameters. This figure illustrates the key operating parameters analyzed in the study.

Figure 9 provides a categorization of various Operating Parameters.

#### Parameters Used Altogether

Model Configurations: The parameters used altogether in model configurations include various elements crucial to the training and performance of the model.

Optimizers such as Adam, SGD, or RMSprop play a significant role in determining how model parameters are updated during training, impacting both the speed of convergence and the final performance of the model [68]. The batch size, which refers to the number of samples processed in a single forward and backward pass, influences the convergence rate and memory requirements, with larger batch sizes generally leading to faster convergence but requiring more memory [68]. Image size, indicating the dimensions of input images processed by the model, affects the level of detail captured and the computational demands, with larger image sizes capturing finer details but necessitating more computational power. Smoothing levels, which determine the degree of smoothing applied to generated frames, are important for reducing artifacts and enhancing visual quality, requiring a balance to maintain realism while minimizing distortions. Finally, the number of training iterations, which refers to how many times the entire dataset is passed through the model, can improve model performance with more iterations but may also lead to overfitting or increased computational costs if excessive.

Audio Parameters: The audio parameters used in talking head generation include Audio Quality and Audio Length. Audio quality refers to the fidelity and clarity of the audio input, which is crucial for generating natural and intelligible speech. High-quality audio enhances the overall realism of the generated content by ensuring that the speech synthesis sounds clear and authentic [69]. Audio length indicates the duration of the audio input. Longer audio segments provide more context and information, which aids in generating coherent and contextually relevant facial expressions and lip movements. This results in more realistic and expressive talking head animations, as the model can better capture the nuances of speech and expression over extended audio inputs. These parameters play a crucial role in ensuring the naturalness, coherence, and realism of the audio–visual synthesis process in talking head generation tasks.

Video Parameters: Video parameters encompass various aspects crucial for generating realistic and expressive talking heads. Facial expression diversity plays a key role, determining the range and variation of facial expressions synthesized by the model, thereby contributing to the expressiveness and realism of the generated faces. Face geometry parameters further enhance realism by controlling various aspects of facial movement and appearance. Gaze coordinates dictate the direction of the subject’s gaze, influencing naturalness and engagement, while facial coefficients and rig parameters regulate facial deformations and movements, ensuring accurate representation. Texture details, encompassing skin tone, texture, and imperfections, enhance authenticity, while lighting settings influence illumination and shadowing, adding depth and dimensionality [68]. Head pose variability ensures natural and dynamic poses, while motion parameters such as frames per second (FPS) affect the smoothness and perceived motion quality of the generated talking heads. Collectively, these parameters contribute to creating compelling and lifelike visual representations in talking head generation.

Table 5 shows the summary of the most important works that have been done in this field using different domains and the most important/major contributions of the research studies in generating talking head images.

## 4. Future Directions

Looking ahead, future research in talking head generation is poised to explore several promising avenues. These advancements will not only refine the perceptual quality and realism of synthesized talking heads but also address computational efficiency, robustness, ethical deployment, and adaptability across diverse real-world applications.

### 4.1. Advancing Multimodal Learning

A key direction in talking head generation research is the continued refinement of multimodal learning approaches to better integrate audio, visual, and textual cues. By leveraging self-supervised and cross-modal learning techniques, future models can achieve more coherent synchronization between speech, facial expressions, and gestures. Additionally, advancements in transformer-based architectures and diffusion models will contribute to enhancing spatiotemporal consistency and long-range dependency modeling in generated videos.

### 4.2. Refining Motion Synthesis and Expression Dynamics

Despite significant progress, current models still struggle with capturing subtle nuances of human expressions, such as micro-expressions, emotional transitions, and spontaneous facial movements. Future work should focus on incorporating higher-resolution facial landmark tracking, biomechanics-inspired facial modeling, and physics-based simulations to improve motion synthesis accuracy. Integrating domain adaptation techniques can further enhance the generalization of expression dynamics across different speakers, languages, and cultural contexts.

### 4.3. Enhancing Model Robustness and Generalization

One major limitation of existing talking head generation models is their vulnerability to data biases, artifacts, and inconsistencies in unseen scenarios. Addressing robustness requires developing more diverse and inclusive datasets that better represent global facial structures, accents, and ethnic variations. Furthermore, adversarial training, meta-learning, and reinforcement learning strategies can be employed to improve adaptability against noise, occlusions, and variations in lighting conditions.

### 4.4. Innovations in Real-Time Processing and Interactive Systems

The integration of talking head generation into real-time applications remains challenging due to computational constraints. Optimizing model architectures through neural acceleration techniques, quantization, pruning, and lightweight network design will be essential for achieving real-time inference on edge devices. Advances in differentiable rendering and neural radiance fields can further enable photorealistic rendering with reduced latency, facilitating interactive virtual assistants, telepresence systems, and immersive environments.

### 4.5. Personalization and Identity Preservation

Personalized talking head generation is an emerging direction with significant implications for virtual assistants, avatars, and digital content creation. Future research should explore identity-preserving generative models capable of maintaining a subject’s unique facial characteristics, speaking style, and behavioral mannerisms across varying contexts. Style transfer and speaker adaptation techniques, supported by large-scale pretrained diffusion and transformer models, can enable controllable and customizable avatar generation while maintaining consistency and authenticity.

### 4.6. Cross-Domain Applications and Multilingual Adaptability

Expanding the applicability of talking head generation to multilingual and culturally diverse scenarios remains an open challenge. Incorporating multilingual speech synthesis and cross-linguistic lip synchronization will enable more inclusive and globally adaptable systems. Additionally, extending research into medical, assistive, and educational domains, such as sign language synthesis and lip-reading assistance, can provide meaningful societal impact.

### 4.7. Lack of Standardized Benchmarks and Unified Evaluation Protocols

Despite rapid progress, the field still lacks standardized benchmark datasets and unified evaluation protocols, limiting fair comparison and reproducibility. Existing studies rely on diverse datasets and task-specific metrics, leading to inconsistent cross-method assessments. Future research should prioritize the development of benchmark datasets that jointly capture audio, video, identity, and emotion under controlled yet diverse conditions. Unified evaluation frameworks integrating visual quality, motion accuracy, audio–visual synchronization, emotional expressiveness, and identity preservation, alongside calibrated human perceptual studies, would significantly enhance comparability, reliability, and real-world relevance.

### 4.8. Ethical Considerations, Misuse Mitigation, and Regulatory Frameworks

As talking head generation systems become increasingly realistic and accessible, they raise significant ethical, legal, and security concerns, particularly regarding identity impersonation, misinformation, and deepfake-based fraud. Addressing these risks requires both detection and prevention strategies, including audio–visual inconsistency analysis, digital watermarking, model fingerprinting, and content provenance tracking. Safeguarding identity rights through informed consent, dataset governance, and technical constraints is equally essential to prevent unauthorized replication. Moreover, responsible deployment demands regulatory oversight, transparency in synthetic content disclosure, and accountability of developers, ensuring that innovation in talking head generation progresses in a trustworthy and sustainable manner.

## 5. Conclusions

The advancements in talking head generation in recent years have been remarkable, driven by cutting-edge deep learning architectures and innovative audio–visual processing techniques. These developments have significantly improved the realism, expressiveness, and interactivity of synthesized facial animations, enabling more immersive user experiences across various domains. Notable progress has been made in lip-sync accuracy, facial expression diversity, and overall visual quality, propelled by advancements in generative models, training methodologies, and dataset utilization.

Despite these achievements, challenges remain in ensuring model robustness, scalability, and real-time processing capabilities. Addressing these limitations requires further integration of multimodal learning approaches, refinement of motion synthesis techniques, and exploration of novel applications across fields such as gaming, virtual assistants, education, and entertainment. This survey contributes to the field by systematically highlighting these challenges, comparing different approaches, and identifying opportunities for novel research directions, thereby providing a unique, cross-domain perspective not fully covered in previous reviews. As the field continues to evolve, breakthroughs in generative AI, real-time processing, and multimodal learning will push the boundaries of realism and interactivity. By tackling these challenges and exploring new directions, researchers can contribute to the development of robust, ethical, and scalable talking head systems, fostering innovation in human-centric AI technologies.

## Figures and Tables

**Table 1:** Comparative Summary of Deep Learning Approaches in THG.

Method Category	Key Strengths	Limitations	Typical Use Case	Realism	Temporal Consistency	Computational Cost	Data Requirements	Generalization Ability
Transformer-based reenactment [38]	Cross-domain flexibility, identity preservation	Sensitive to extreme poses	Cartoon-to-real transfer	Moderate	Moderate	Moderate	High (multi-domain data)	High across domains
3D reconstruction-based models [30]	Geometric consistency, robustness	High computational cost	High-fidelity synthesis	High	High	High	High (3D supervision or multi-view)	Moderate
One-shot generation [26]	Minimal reference data	Limited pose diversity	Personalized avatars	Moderate–High	Moderate	Moderate	Low	Low–Moderate
GAN-based models [11]	High visual realism	Training instability	Photo-realistic avatars	High	Low–Moderate	High	High	Moderate
Motion-aware RNNs [39]	Natural head motion	Limited texture detail	Speech-driven animation	Moderate	High	Low–Moderate	Moderate	High
Attention-based models [16]	Strong audio–visual synchronization	Data-intensive training	Multimodal synthesis	High	High	High	High	Moderate–High

**Table 2:** Various datasets and their salient statistics.

Dataset Name	Type	Data Volume	Subjects/Duration Units	Image Available	Obvious Head Movements	Collection Environment
100STYLE	Image	4,000,000 frames	100 subjects	No	No	Motion capture studio
LSP	Image	2000 images	–	Yes	Yes	Flickr (images)
VOCASET	Image	29 min (60 fps)	12 subjects	Yes	Yes	Standardized phonetic protocol
BIWI	Image	15,000 images	20 subjects	Yes	Yes	Automotive setup (Kinect)
UvA-NEMO	Video	1240 smile videos	400 subjects	Yes	Yes	Controlled lab environment
TED-Talks	Video	3035 videos	–	Yes	Yes	TED stage recordings
CelebV-HQ	Video	35,666 video clips	15,653 subjects	Yes	Yes	YouTube interviews
Tai-Chi-HD	Video	250 videos	–	Yes	Yes	Controlled environment
MEAD	Audio–visual	40 h	60 subjects	Yes	Yes	Controlled lab environment
GRID	Audio–visual	27.5 h	34 subjects	No	No	Controlled lab environment
LRW	Audio–visual	173 h	1000+ subjects	Yes	Yes	BBC (TV/interviews)
TSG Zero-EGGS	Audio–visual	67 sequences	1 subject	Yes	Yes	Controlled environment
Motorica Dance	Audio–visual	6.0 h	8 subjects	Yes	Yes	Motion capture studio
3D-VTFSET	Audio–visual	20.0 h	300 subjects	Yes	Yes	YouTube videos
TCD-TIMIT	Audio–visual	6913 sentences	62 subjects	Yes	Yes	Controlled lab environment
CREMA-D	Audio–visual	11.1 h	91 subjects	No	No	Controlled lab environment
LRS/LRS3	Audio–visual	438 h	5000+ subjects	Yes	Yes	TED/YouTube
HDTF	Audio–visual	15.8 h	362 subjects	Yes	Yes	High-resolution video
VoxCeleb2	Audio–visual	2400+ h	6112 subjects	Yes	Yes	YouTube interviews
RAVDESS	Audio–visual	7356 speeches/songs	24 subjects	Yes	Yes	Controlled emotional recording

**Table 3:** Tools/Algorithms and corresponding datasets.

Tool/Algorithm	Dataset
GANs	VoxCeleb1, CelebV
Flow2Flow	CelebV-HQ, VoxCeleb2
Audio2head	VoxCeleb, GRID, LRW
SadTalker	VoxCeleb, HDTF
VividTalk	HDTF, VoxCeleb
CVTHead	VoxCeleb1, VoxCeleb2
HeadGAN	VoxCeleb
ToonTalker	VoxCeleb, CelebA-HQ

**Table 4:** Limitations and inconsistencies of commonly used metrics.

Metric	Evaluates	Strengths	Limitations
FID	Image realism	Captures distribution-level realism	Sensitive to dataset size; ignores temporal consistency
PSNR	Pixel similarity	Simple and interpretable	Poor correlation with perceptual quality
SSIM	Structural similarity	Captures luminance and structure	Favors smooth outputs
LPIPS	Perceptual similarity	Aligns well with human perception	Computationally expensive
CPBD	Sharpness	Models human blur perception	Ignores motion coherence
LMD	Lip-sync accuracy	Accurate spatial lip alignment	Depends on landmark detection quality
LLVE	Temporal lip motion	Captures motion smoothness	Sensitive to frame noise
MCD	Audio quality	Measures spectral similarity	Ignores prosody and emotion
CSIM	Identity preservation	Maintains identity consistency	Does not ensure expression realism

**Table 5:** Comparison of Various Models and Their Contributions Over Time.

Model	Year	Driving Modality	Generative Mechanism	Key Intermediate Representation	Strengths	Limitations
Speech-Driven 3D Face Animation with Composite and Regional Facial Movements	2023	Audio	Parametric + Regression	3D mesh regions	Fine-grained regional control	Requires high-quality 3D data
Everybody’s Talkin: Let Me Talk as You Want	2022	Audio	Neural Rendering	Latent motion codes	Flexible speaking style control	Limited explicit geometry modeling
A Morphable Model for the Synthesis of 3D Faces	2023	Audio	3D Parametric Model	3DMM coefficients	Strong geometric consistency	Less expressive fine details
FaceComposer	2024	Audio/Text	Unified Generative Model	Disentangled latent factors	Versatile multi-task generation	High model complexity
LipSync3D	2021	Audio	Regression-based	Normalized pose & lighting parameters	Data-efficient personalization	Limited expressiveness
ADNeRF	2021	Audio	Neural Radiance Fields	NeRF density & color fields	High photorealism	Computationally expensive
Audio-Driven 3D Face Animation	2022	Audio	Parametric + Neural	3D facial parameters	Stable lip-sync	Limited stylistic diversity
StyleTalk	2023	Audio	GAN-based	Style and motion embeddings	One-shot style control	Sensitive to pose variation
CVTHead	2024	Audio	Transformer-based	Vertex feature embeddings	Precise geometric control	Heavy training requirements
MODA	2023	Audio	Attention-based	Dual attention maps	Efficient one-shot animation	Moderate visual realism
ToonTalker	2023	Audio	Transformer-based	Cross-domain latent features	Strong domain transfer	Cartoon-to-real gap sensitivity
DiffPoseTalk	2023	Audio	Diffusion Models	3D pose & expression latents	Diverse and natural motion	Higher inference latency
VLOGGER	2024	Audio/Image	Diffusion Models	Spatiotemporal latent maps	Strong temporal consistency	Not real-time
DREAM-Talk	2023	Audio	Two-stage Diffusion	Emotion & lip refinement codes	Emotionally expressive output	Computational overhead
VividTalk	2024	Audio	Two-stage Hybrid	Head pose & mouth disentanglement	Accurate synchronization	Requires strong priors
Depth-Aware GAN	2022	Audio	GAN-based	3D-aware depth features	Improved realism	Requires 3D preprocessing
Audio2Head	2021	Audio	Modular Neural Framework	Head pose & expression vectors	Natural head motion	Complex pipeline
InstructNeuralTalker	2023	Audio/Text	NeRF + Instruction Learning	Editable radiance fields	Interactive editing	Heavy training cost
SadTalker	2023	Audio	3D Motion Learning	3D motion coefficients	High visual quality	Identity drift over long videos
HeadGAN	2021	Audio	GAN-based	Latent identity embeddings	One-shot synthesis	Temporal inconsistency under large pose variation

## Data Availability

No new data were created or analyzed in this study. Data sharing is not applicable to this article.
